# Rural–Urban Differences in Hypertension Prevalence and Control in a Large Regional Health System Cohort

**DOI:** 10.3390/jcm15114233

**Published:** 2026-05-30

**Authors:** Yordanos M. Tiruneh, Susan McBride, Huaxin Song, Matthew Decaro, Alaa Rihan, Theresa Byrd, Alex Baham, Jared W. Magnani, Elmer V. Bernstam, Jarett D. Berry

**Affiliations:** 1 School of Medicine, The University of Texas at Tyler, Tyler, TX 75799, USA; 2 Division of Infectious Diseases, Department of Medicine, The University of Texas Southwestern Medical Center, Dallas, TX 75390, USA; 3 Institute for Health Innovations, Data Science, and Research, The University of Texas at Tyler, Tyler, TX 75799, USA; 4 School of Nursing, The University of Texas at Tyler, Tyler, TX 75799, USA; 5 D. Bradley McWilliams School of Biomedical Informatics, The University of Texas Health Science Center Houston, Houston, TX 77030, USA; 6 Tyler Family Circle of Care, Tyler, TX 75702, USA; 7 School of Health Professions, The University of Texas at Tyler, Tyler, TX 75708, USA; 8 Department of Medicine, University of Pittsburgh, Pittsburgh, PA 15261, USA; 9 Division of General Internal Medicine, McGovern Medical School, The University of Texas Health Science Center Houston, Houston, TX 77030, USA

**Keywords:** hypertension, rural health, rural–urban differences, blood pressure control, cardiovascular disease prevention, Texas

## Abstract

**Background:** Rural US populations bear a high burden of cardiovascular disease, with hypertension contributing to the rural–urban gap. We examined hypertension prevalence and control across the rural–urban continuum among patients in a large healthcare system in Northeast Texas. **Methods:** We analyzed the electronic health record data from 363,539 patients with at least one outpatient encounter between September 2021 and April 2025. We defined hypertension using ICD-10-CM codes, antihypertensive prescriptions, or a blood pressure ≥ 130/80 mm Hg. We classified rurality using the Rural–Urban Commuting Area categories. Multivariable logistic regression models were adjusted for age, sex, race, ethnicity, insurance, and level of engagement with the healthcare system. **Results:** Compared with metropolitan areas, micropolitan residents had lower odds of hypertension (OR = 0.85). We found uncontrolled hypertension also to be more common in metropolitan areas, with a lower prevalence observed across non-metropolitan categories, particularly among isolated rural residents (OR = 0.82). Black patients exhibited higher rates of uncontrolled hypertension across all rural–urban categories. Continuity of care was associated with hypertension prevalence but not control. **Conclusions:** Hypertension prevalence and control vary across the rural–urban continuum in Northeast Texas, with consistent differences observed between metropolitan and some non-metropolitan residents. Persistent racial differences across all geographic settings support the need for targeted prevention and management strategies.

## 1. Introduction

Rural populations in the United States suffer from disproportionately higher cardiovascular disease (CVD) morbidity and mortality than those residing in urban areas [1,2,3,4]. This gap reflects a greater burden of uncontrolled risk factors and limited access to health care facilities in rural communities [5,6,7]. Hypertension, a leading yet modifiable CVD risk factor, largely drives this gap [8,9,10]. National data show a greater hypertension prevalence in rural areas than urban counties, where estimates rise as high as 40% in rural areas compared with 29% in urban areas [11].

Even when they can access effective antihypertensive treatments, rural residents find it more difficult to achieve blood pressure control, and often face barriers to hypertension care [1]. Rural populations experience limited access to outpatient hypertension services, contributing to gaps in preventive and long-term disease management [4,12]. Social and structural factors, including limited access to care, lower incomes, transportation barriers, long travel distances, and fewer local preventive-care resources determine the extent of and success in managing hypertension in rural communities [13,14,15,16]. Most studies have relied on a binary rural/urban classification system that masks variations in geographic isolation across communities [17].

Rural–urban differences in CVD are especially pronounced in the Southern states [18,19], a pattern we also observe in Texas. Rural Texans experience higher CVD mortality, with one county-level analysis finding higher age-adjusted rates than in metropolitan areas (750.9 vs. 659.4 per 100,000 population, 1999–2019) [20]. In medically underserved regions, such as Northeast Texas, a 35-county area with high cardiovascular mortality and stroke burdens, these pronounced disparities call for research attention [21]. The region’s CVD hospitalization rates exceed the state average, and risk factors such as poor diet, smoking, and physical inactivity are common [22,23]. Data on hypertension identification and management across the rural–urban continuum in this region are, however, limited.

In this study, we used electronic health record (EHR) data from a large regional health system to examine hypertension across rural and urban communities in Northeast Texas. We quantified hypertension prevalence along the rural–urban continuum and evaluated the extent to which blood pressure is controlled among patients receiving treatment. This approach makes a detailed assessment of diagnosis and management patterns in a large patient population possible. We hypothesize that differences in healthcare access and geographic isolation reflect the variations in hypertension prevalence and control across rural–urban settings.

## 2. Materials and Methods

### 2.1. Data Source and Study Population

We analyzed data from a large healthcare system and physician practices in Northeast Texas from September 2021 to April 2025. We constructed our cohort of unique individuals (≥18 years) with at least one recorded outpatient encounter on the electronic health record (EHR).

### 2.2. Outcomes

The primary outcomes of interest were hypertension and uncontrolled hypertension. We defined hypertension using a pragmatic EHR-based phenotype that applied one of the following three criteria during the study period: (1) an ICD-10 diagnosis code for hypertension; (2) an active prescription for antihypertensive medications; and (3) an outpatient blood pressure (BP) ≥ 130 mm Hg systolic or ≥80 mm Hg diastolic [24]. We limited the blood pressure measurements to outpatient encounters, excluding emergency department (ED) visit records. We assessed uncontrolled hypertension among patients prescribed at least one antihypertensive medication and at least one available BP measurement during follow-up, yielding a denominator of 45,642 patients. We defined uncontrolled hypertension primarily by reference to an individual’s most recent reading of BP ≥ 130 mm Hg systolic or ≥80 mm Hg diastolic.

### 2.3. Exposure Variable—Rurality

We characterized rurality using the Rural–Urban Commuting Area codes, as metropolitan (RUCA 1–3), micropolitan (RUCA 4–6), small-town (RUCA 7–9), and isolated-rural (RUCA 10) areas based on the patients’ residential locations [25].

### 2.4. Covariates

Patient-level covariates included age, sex (male, female), race (White, Asian, Black, Other), ethnicity (Hispanic, non-Hispanic), and primary insurance classification (private, Medicare, Medicaid, “Other”, and “Missing”). Race and Ethnicity were captured as administrative and self-reported categories obtained from electronic health records and were not intended to represent biological classifications [26]. We measured engagement with the healthcare system using two variables, annual encounter rate and continuity of care. We defined the annual encounter rate as a continuous measure calculated as the total number of outpatient visits divided by the total follow-up time in years, measured starting with the first and ending with the last qualifying visit. Continuity of care was defined as a binary variable, categorized as ‘yes’ if the patients had at least one encounter in each of the years 2022, 2023, and 2024, and ‘no’ if otherwise. We excluded the years 2021 and 2025 from this definition because the encounter data for those years were incomplete. Encounters were defined as any qualifying patient-facing interactions documented in the EHR.

### 2.5. Statistical Analysis

We used multivariable logistic regression models with a binomial link (*glm()*) to assess associations between rurality and the following two outcomes: (1) hypertension prevalence and (2) percentage of uncontrolled hypertension. All multivariable models were adjusted for age, sex, race, ethnicity, insurance type, primary language, insurance type, annual encounter rate, and continuity of care. We modeled geographic classification as a four-level categorical variable, with metropolitan as the reference category. Estimates and confidence intervals were pooled, and Rubin’s rules were applied via the *pool()* function. To optimize model fit, we excluded non-contributory covariates. We treated two-sided *p* < 0.05 as statistically significant, and reported adjusted odds ratios with 95% confidence intervals. We conducted all analyses using R (v4.3.2).

To address the missing race data (17% of cases), we made multiple imputations using the mice package in R (v4.3.2). Five datasets were imputed using the polyreg method. We included ethnicity, age, and insurance type in the imputation model for race, with ethnicity weighted highest for the imputation. Although we found considerable missingness in ethnicity and insurance data, we retained both in their original forms, with “unknown” or missing values coded as separate categories in the analyses rather than imputed, as these values likely reflect non-random reporting or administrative coding practices rather than random missingness.

To assess the robustness of our findings, we conducted sensitivity analyses using alternative definitions of hypertension phenotypes along two dimensions, BP threshold (≥130/80 vs. ≥140/90) and measurement approach (single most recent reading vs. the average of the three most recent readings occurring within a rolling 12-month window). This technique yielded four phenotype definitions. We excluded those patients lacking three BP readings within any 12-month window. We applied these alternative definitions in both the hypertension prevalence and uncontrolled hypertension analyses.

We used an Elicit AI tool (https://elicit.com) to identify and review the rural–urban comparative studies on hypertension. The synthesis and interpretation of the literature were conducted by the authors.

## 3. Results

### 3.1. Baseline Characteristics

Table 1 presents the distribution of the study population (n = 363,539) stratified by the RUCA codes, and categorized as metropolitan, micropolitan, small-town, or isolated-rural areas. The proportion of adults > 65 years old was higher in non-metropolitan regions (rising to 44%). Women comprised 54% of the overall patient population, with slightly fewer in isolated rural areas (52%). The majority of patients were White (73%), with higher concentrations in isolated rural areas (83%).

Insurance classification differed based on rurality (*p* < 0.001). We observed private coverage more commonly in metropolitan areas, while Medicare coverage became steadily more common as the observations moved from metropolitan to isolated areas, with higher Medicare reliance in non-metro areas. High levels of missing or unknown insurance data (35%) likely reflect documentation gaps or lack of coverage across all categories. Continuity of care varied modestly across the rural strata (*p* < 0.001). The median annual encounter rate was nine visits, with rurality making no difference (*p* = 0.39).

### 3.2. Hypertension Prevalence

Mean blood pressure levels were similar across the geographic areas. In the multivariable-adjusted model, hypertension prevalence was higher in older adults compared with patients <50 years (OR = 1.94, 95%CI: 1.90–1.97 for ages 50–65; OR = 2.35, 95%CI: 2.30–2.40 for ≥65+, *p* < 0.001), males (OR = 1.34, 95%CI: 1.32–1.36, *p* < 0.001), and non-Hispanic patients (OR = 1.29, 95%CI: 1.26–1.33, *p* < 0.001). Compared with Whites, Black patients had a slightly higher prevalence (OR = 1.03, 95%CI: 1.00–1.05), while other racial groups showed no association.

**Figure 1:**
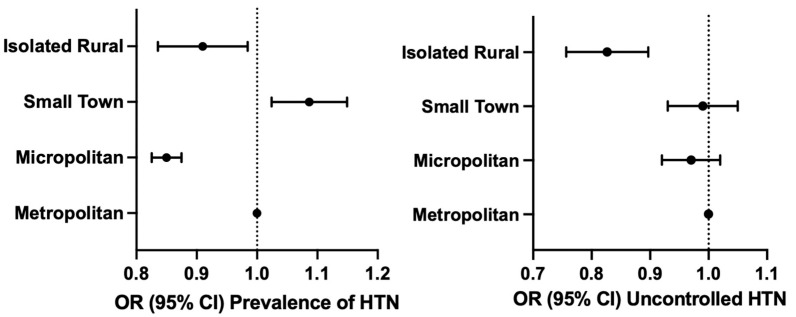
Adjusted Odds Ratios for Hypertension Prevalence and Control by Rurality (N = 45,642).

Hypertension prevalence differed by insurance type, with lower odds among Medicaid and “Other”/missing insurance groups and higher odds among Medicare beneficiaries. Rurality showed mixed effects (Figure 1) after adjusting for other risk factors. Compared with metropolitan areas, micropolitan (OR = 0.85, 95%CI: 0.84–0.86, *p* < 0.001) and isolated-rural areas (OR = 0.91, 95%CI: 0.88–0.94, *p* < 0.001) faced a lower hypertension risk, while those in small-towns faced a greater risk (OR = 1.09, 95%CI: 1.06–1.11, *p* < 0.001). Continuity of care was strongly associated with a hypertension diagnosis (OR = 3.35, 95% CI: 3.30–3.41, *p* < 0.001).

### 3.3. Uncontrolled Hypertension

In the adjusted model (N = 45,642), older age was associated with better blood pressure control, with lower odds of uncontrolled hypertension among those aged 50–65 years (OR = 0.87, 95%CI: 0.81–0.93, *p* < 0.001) and ≥65 years (OR = 0.70, 95%CI: 0.65–0.75, *p* < 0.001), compared with patients under 50. Males faced greater risk than females (OR = 1.15, 95%CI: 1.11–1.20, *p* < 0.001) and Black patients had a significantly higher risk (OR = 1.39, 95%CI: 1.31–1.48, *p* < 0.001), while Asian (OR = 0.70, 95%CI: 0.57–0.85, *p* = 0.0026), or “Other” racial groups (OR = 0.69, 95%CI: 0.49–0.99, *p* = 0.0302) faced a lower risk relative to White. Ethnicity showed no significant association.

Medicare beneficiaries showed a significantly lower risk of uncontrolled hypertension (OR = 0.89, 95%CI: 0.84–0.94, *p* < 0.001), while patients with missing insurance data had a greater risk (OR = 1.16, 95%CI: 1.04–1.30, *p* = 0.0028). Patients from isolated rural areas faced a significantly lower risk (Figure 1) of exhibiting uncontrolled hypertension (OR = 0.82, 95%CI: 0.76–0.90, *p* < 0.001) than those in metropolitan areas. In contrast to a diagnosis of hypertension, continuity of care was not significantly associated with uncontrolled hypertension. The annual encounter rate was marginally but significantly associated with effective hypertension control (*p* < 0.001).

Across sensitivity analyses using alternative definitions of hypertension, we found that the factors associated with hypertension had large effect sizes and were stable across the models, while associations with uncontrolled hypertension were smaller and more variable. We found age to be consistently associated with higher odds of hypertension prevalence but lower odds of uncontrolled hypertension, and female sex was associated with lower odds for both outcomes in all analyses. We also found consistent association between Black race and higher odds of prevalence and uncontrolled hypertension across all models, with a stronger effect on uncontrolled hypertension. Across all models, continuity of care remained strongly associated with hypertension prevalence, but not with uncontrolled hypertension. Rurality varied in magnitude and significance across the models for prevalence, but we found a more consistent association with lower uncontrolled hypertension in categories reflecting greater rurality, particularly in small-town and isolated rural areas. Insurance effects were stable for hypertension prevalence, but with uncontrolled hypertension these effects varied across the models in both direction and magnitude.

## 4. Discussion

This analysis identified three key findings. Hypertension prevalence differed across rural settings of Northeast Texas, with lower rates in isolated rural and micropolitan areas and higher rates in small towns. We observed that poor hypertension control occurred more often in metropolitan patients. Racial differences in hypertension control persisted across the geographic regions.

Although national datasets [27] report higher rural hypertension prevalence, (e.g., NHANES: 48.6% in rural vs. 45.4% in medium–small and 41.1% in large metropolitan areas) [28], our data showed a lower recorded diagnosis in rural patients. This pattern may reflect delayed care-seeking and/or limited access to continuous or preventive services among rural patients in our healthcare system [29,30,31]. Using alternative definitions of hypertension in sensitivity analyses, we found that the direction of rural–urban differences was generally preserved with similar directional patterns but varied effect sizes across the definitions, especially in the small-town and isolated rural categories. These findings suggest that the observed differences are heterogeneous, perhaps influenced by definitions of hypertension rather than the true underlying disease burden.

In our data, rural patients were less likely to have uncontrolled hypertension than their metropolitan counterparts. This finding is consistent with the REGARDS study, which also found no rural disadvantage in hypertension control among those on antihypertensive medications [32]. We cannot, however, rule out selection bias, as rural patients captured within the healthcare system may represent a more engaged subset of patients. Alternatively, sicker rural patients with poorly controlled hypertension may disproportionately present to emergency or inpatient settings and may be missed in outpatient data. These findings indicate that data-source choices and phenotype specifications influence interpretations and underscore the importance of understanding patient–population characteristics.

We found that continuity of care varied modestly but significantly by rural–urban classification. The limited variation observed in continuity of care should be interpreted in light of the fact that engagement in care reflects a combination of interpersonal continuity and structural constraints [33,34]. In areas with fewer provider options (e.g., isolated rural areas), “continuity of care” may reflect limited choice rather than stronger patient–clinician relationships. In metropolitan areas, care across multiple providers and healthcare systems likely lowers the measured continuity of care within a single network. The modest differences we found in continuity of care and the inconsistent association with uncontrolled hypertension across sensitivity analyses suggest that the observed differences in BP control cannot be explained by reference to continuity of care alone.

We found broadly similar annual outpatient encounter rates across metropolitan and non-metropolitan settings, but overall visit counts alone may not capture the differences in utilization. Rural patients likely receive more complex outpatient visits (receiving medically complex services within the same visit) or rely more heavily on emergency or inpatient services not captured as primary care encounters. In addition, variation in patient complexity could mask access-related differences if patients with severe illness require more frequent care. Future studies incorporating inpatient and emergency visits and adjusting for comorbidities would provide a more accurate assessment of disease burden and service use in rural populations.

Our findings show substantial variation across rural populations. In our cohort, rural patients were generally older, more likely to be White, and depended more heavily on Medicare. Nonetheless, consistent with national evidence of persistent racial differences in hypertension control [35,36], Black patients show higher rates of uncontrolled hypertension across the regions. These outcome differences reflect limited healthcare access, lower income and education, environmental stressors, and geographic isolation, which can hinder effective hypertension management [13,14,15,37]. The relationship between cardiometabolic risk factors, cardiovascular outcomes, and social conditions is well established [10]. Prior work has also revealed rural–urban gaps in cardiometabolic risk, and a higher hypertension burden among Black individuals [38].

We recognize that reducing CVD risk comprehensively requires a broader approach. Advanced cardiac imaging, such as global longitudinal strain metrics, offers superior prognostic value for identifying high-risk hypertensive patients that exceeds blood pressure monitoring alone [39,40,41,42]. Integrating these approaches into clinical practice may improve risk stratification and facilitate personalized management for patients with uncontrolled hypertension, especially in rural settings with limited access to specialty care; however, access, cost, and feasibility remain important challenges. These developments warrant additional research to understand how these advanced tools can be used effectively in routine care across large healthcare systems and underserved rural communities.

This study is subject to several limitations. First, we analyzed outpatient EHR data from a single health system. Consequently, our findings reflect those patients captured within this system rather than broader population-based estimates. The observed rural–urban differences might therefore reflect variations in healthcare access and utilization in addition to disease burden. Second, we excluded inpatient and emergency encounters, potentially limiting interpretation, as rural patients may rely more on acute care settings. Third, EHR data lacked key socioeconomic measures, and unmeasured factors, including transportation barriers, health literacy, and local health-system organization, likely influenced both engagement in care and encounter frequency. Fourth, our definition of hypertension (based on diagnostic codes, antihypertensive medication use, and an elevated BP readings) classified patients as hypertensive if they met any one of the criteria we used. This approach, while feasible for a longitudinal cohort, may capture levels of diagnostic certainty or clinical context depending on the criterion met. Sensitivity analyses revealed estimates that varied across alternative definitions, albeit in overall consistent patterns, indicating that our results might have been influenced by phenotype specification. Despite these limitations, however, the large sample size and detailed patient-level data shed light on the rural–urban continuum of hypertension prevalence and control in a diverse patient population.

## 5. Conclusions

This study examined hypertension prevalence and control across urban and rural areas in Northeast Texas using the RUCA codes and patient-level factors. We observed differences in hypertension prevalence across rural–urban categories, higher rates of uncontrolled hypertension in metropolitan areas, and persistent differences in hypertension control rates by patient race along the entire rural–urban continuum. Differences across rural categories may reflect variations in healthcare access and utilization behavior, in addition to the underlying disease burden. These findings reflect health system-based patterns and not necessarily population-based estimates.

## Figures and Tables

**Table 1:** Baseline characteristics of patients in the health system by RUCA designated Rural–Urban Residence.

	Overall	Metropolitan	Micropolitan	Small Town	Isolated Rural	*p*-Value
		N (%) (Unless otherwise indicated)				
	363,539	187,479 (52)	113,157 (31)	43,594 (12)	19,309 (5)	
**Age Group**						<0.001
*<50*	156,626 (43)	84,781 (45)	47,915 (42)	17,714 (41)	6216 (32)	
*50–64*	86,898 (24)	44,345 (24)	26,659 (25)	10,334 (24)	4560 (24)	
*65+*	120,015 (33)	58,353 (31)	37,583 (33)	15,546 (36)	8533 (44)	
*Missing*	0	0	0	0	0	
**Sex**						<0.001
*Female*	197,905 (54)	102,236 (54)	61,826 (55)	23,758 (55)	10,085 (52)	
*Male*	165,456 (46)	85,116 (46)	51,293 (45)	19,829 (45)	9218 (48)	
*Missing*	178 (<1)	127 (<1)	38 (<1)	7 (<1)	6 (<1)	
**Race**						<0.001
*White*	264,955 (73)	134,663 (72)	81,126 (72)	33,105 (76)	16,061 (83)	
*Asian*	2918 (1)	2225 (1)	435 (<1)	193 (<1)	65 (<1)	
*Black*	30,778 (8)	16,512 (9)	9215 (8)	4220 (10)	831 (4)	
*Other*	778 (<1)	450 (<1)	189 (<1)	105 (<1)	34 (<1)	
*Missing*	64,110 (18)	33,629 (18)	22,192 (20)	5971 (14)	2318 (12)	
**Ethnicity**						<0.001
*Hispanic*	34,492 (9)	16,766 (9)	13,432 (12)	3474 (8)	820 (4)	
*Non-Hispanic*	275,277 (76)	141,603 (76)	83,807 (74)	34,258 (79)	15,609 (81)	
*Missing*	53,770 (15)	29,110 (15)	15,918 (14)	5862 (13)	2880 (15)	
**Insurance**						<0.001
*Private*	126,292 (35)	69,452 (37)	35,631 (32)	15,253 (35)	5956 (31)	
*Medicaid*	21,951 (6)	10,151 (5)	7922 (7)	2899 (7)	979 (5)	
*Medicare*	85,985 (24)	41,815 (22)	26,879 (24)	11,324 (26)	5967 (31)	
*Missing/Unknown*	126,066 (35)	64,422 (35)	41,525 (36)	13,838 (32)	6281 (33)	
*Other (Indigenous)*	3245 (1)	1639 (1)	1200 (1)	280 (<1)	126 (<1)	
**Engagement with Care**						
Continuity of Care *	127,444 (35)	67,410 (36)	36,968 (33)	16,250 (37)	6816 (35)	<0.001
*Average annual encounter* Median (IQR)	9 (19)	9 (21)	8 (17)	10 (20)	10 (21)	0.39
**Vitals**						
*Mean Systolic BP*	127.7 (16.9)	127.8 (17.0)	128.0 (17.3)	127.2 (16.0)	127.4 (16.3)	0.56
*Mean Diastolic BP*	76.1 (10.6)	76.0 (10.6)	76.4 (10.7)	76.4 (10.3)	74.9 (10.2)	0.07

* Continuity of care is defined as having at least one encounter per year during the study period (2022–2024).

## Data Availability

The dataset generated and or analyzed in this study are not publicly available because they include patient level electronic health record information and are restricted by institutional policy and privacy regulations. Data access requests may be considered by the corresponding author pending institutional approval and appropriate data use agreement.

## Abbreviations

The following abbreviations are used in this manuscript:

CVD	Cardiovascular Disease
BP	Blood Pressure
EHR	Electronic Health Record
ICD-10-CM	International Classification of Diseases, Tenth Revision, Clinical Modification
ED	Emergency Department
RUCA	Rural–Urban Commuting Area
